# A cross-sectional retrospective and prospective service evaluation of head and neck cancer patients’ swallowing and quality of life concerns: are we doing enough?

**DOI:** 10.1007/s00520-026-10906-5

**Published:** 2026-06-23

**Authors:** Sinead Rothrie, Grainne Brady, Justin Roe

**Affiliations:** 1 Department of Speech, Voice and Swallowing, The Royal Marsden NHS Foundation Trust, London, UK; 2 Department of Surgery and Cancer, Imperial College London, London, UK

**Keywords:** Head and neck cancer, Survivorship, Quality of life, Dysphagia, Swallowing toxicity, Speech and language therapy

## Abstract

**Introduction:**

Head and neck cancer (HNC) treatment causes significant functional sequelae including changes to eating, drinking and swallowing. Long-term surveillance currently focuses on the potential identification of disease recurrence yet does not seek to address ongoing quality of life (QoL). We sought to evaluate the potential unmet needs of patients undergoing routine surveillance following curative treatment for primary HNC at a tertiary referral centre with a particular focus on swallowing toxicity.

**Methods:**

A cross-sectional *retrospective* and *prospective* evaluation was undertaken at a single tertiary referral centre. Patient medical records were reviewed retrospectively over a 3-month period to ascertain if routine surveillance consultations addressed any potential swallowing-related toxicity. Unmet needs were prospectively measured using the Patients Concerns Inventory for Head and Neck Cancer Post Treatment (PCI-HaNC-PT).

**Results:**

The *retrospective* sample included 52 patients where data was collected via medical note review. There was documented evidence of surveillance for swallowing-related toxicity in 13% (*n* = 7) of cases. Of the seven patients questioned about any potential swallowing-related toxicity, six reported difficulties with only one referred for formal assessment to speech and language therapy (SLT). The *prospective* sample included 74 individuals. On screening for potential unmet needs using the PCI-HaNC-PT, 78% (*n* = 58) reported at least one unmet QoL concern. The most commonly reported concerns included dry mouth 41% (*n* = 30), difficulty swallowing 27% (*n* = 20), fear of recurrence 20% (*n* = 15), head and neck pain 18% (*n* = 13) and difficulties chewing 16% (*n* = 12).

**Conclusion:**

These data demonstrate that swallowing toxicity following treatment was not routinely elicited during surveillance appointments. Patients presented with QoL-related concerns in the post treatment setting with eating and drinking difficulties being most prevalent. More detailed consideration is needed to understand how patients’ needs can be better identified to enhance and improve ongoing QoL during the surveillance period following treatment.

**Implications for Cancer Survivors:**

• Head and neck cancer treatment causes significant long-term changes to many patients’ functional outcomes including eating and drinking.

• Within this evaluation routine surveillance models did not provide sufficient assessment of patients’ ongoing QoL concerns following treatment end.

• These data demonstrate swallowing toxicity following treatment was not routinely elicited during surveillance appointments.

• The utilisation of holistic prompts list such as PCI-HaNC-PT can provide opportunities for patients to share their experiences and help shape a personalised response from treatment care teams.

## Introduction

Head and neck cancers (HNC) are malignant tumours of the upper aerodigestive tract. The incidence of HNC continues to rise worldwide [1]. Risk factors historically include smoking and alcohol consumption [2]. However, there has also been an increase in human papilloma virus (HPV)-related disease [3], particularly in the oropharynx [4], predominately within the Western hemisphere [5].

Approximately 13,000 patients are treated for HNC by the NHS every year [6]. The curative treatment pathway uses (chemo)radiation, surgery or a combination of these treatments [7]. These treatments, specifically when used in combination, are known to result in profound side effects with both short term and/or chronic symptoms [8, 9].

Swallowing difficulties (dysphagia) is one of the most debilitating side-effects of HNC and/or its treatment [10] and can have a major impact on a patient’s overall quality of life (QoL) [11]. While dysphagia is important, there are a myriad of related symptoms which impact a patient’s ability to eat and drink post treatment including difficulty chewing, mouth dryness (xerostomia) and pain within the head and neck [8]. This combination of symptoms is known as swallowing-related toxicity and we will refer to it as such in this paper. It can occur as an acute, persisting or late effect of treatment, and speech and language therapists (SLTs) are acknowledged as the experts in this field, providing assessment, support and interventions for patients [12].

There is a growing body of evidence which focuses particularly on the impact of late radiation-associated dysphagia (late-RAD) [13–16]. While the definitions and terminology of late-RAD continue to be clarified, many agree that it is a new deterioration or decline in swallowing function, following a period of stability after treatment completion.

While the understanding of late RAD and the significant after effects of HNC treatment is increasing [17], there is also an increased drive to review current models of care after treatment, including HNC surveillance and monitoring. Current standard of care (SOC) within the UK specifies consultant-led 5-year follow-up. This model of care is increasingly under scrutiny. Evidence has suggested that many recurrences are identified outside of the pre-scheduled routine surveillance appointments [18]. Moreover, with rising survival rates, this model of care is becoming increasingly unsustainable, with demand outstripping capacity.

A current randomised controlled study in the UK is seeking to compare existing SOC to a patient-initiated follow-up protocol. This envisions patients having a PET-CT scan at 12 months to determine cancer recurrence, followed by patients being able to self-report red flag symptoms to an open referral system [19]. Furthermore, ongoing advancements in biomarker technologies suggest there may soon be more specific and personalised methods of follow-up care to accurately detect recurrent or residual disease [20].

Both of these new and innovative pathways are hugely valuable; however, it is also the case that HNC patients are a heterogeneous group with known disparities in socioeconomic status and health system access. This heterogeneity is known to lead to later staged diagnosis of disease and poorer survival outcomes, particularly in the first 12–18 months after diagnosis [21–23]. As models of monitoring become more focused on the onus of the individual to alert the system, this may increase disparity of need in populations who already experience inequity.

Finally, established literature also demonstrates a link between dysphagia, aspiration and pneumonia as well as non-cancer morbidity in those treated for HNC, [24, 25] and the ruling out of recurrence alone potentially does not continue to meet patient’s overall healthcare needs.

This cross-sectional study aimed to identify potential unmet needs of patients who are under routine surveillance following treatment for primary HNC with a particular focus on swallowing toxicity. We hypothesised that swallowing-related toxicity is currently under elicited during routine surveillance. This study sought to understand both whether patients were routinely asked about dysphagia or related difficulties and whether they independently identified ongoing unmet QoL concerns linked to their cancer treatment.

## Materials and methods

### Design

A cross-sectional evaluation was undertaken with both *retrospective* and *prospective* data collection at a single tertiary referral centre (The Royal Marsden NHS Foundation Trust).

*Retrospective* data collection took place via case note review. HNC outpatient consultant-led medical clinic lists were reviewed by an SLT and the number of patients under surveillance follow-up was recorded. Data was reviewed from a 3-month period between July and September 2023. Case notes were reviewed to identify if patients who were under active surveillance and were seen for routine review during that period were (1) asked about any potential swallowing-related toxicity and (2) if any onward referrals were initiated based on their reported swallowing status. Data extracted was inputted into a password-protected Excel spreadsheet with authors SR and GB agreeing on terms to be included.

The *prospective* arm sought to identify unmet needs/concerns using a validated patient-reported item prompt list known as the Patient Concerns Inventory for Head and Neck Cancer Post Treatment (PCI-HaNC-PT) [26]. Eligible patients who were attending routine surveillance HNC appointments between October 2023 and December 2023 were asked whether they would like to take part in the completion of the PCI-HaNC-PT.

The PCI-HaNC-PT [26] is a condition-specific prompt list designed to be completed by patients prior to their consultation to ensure that issues related to physical or mental well-being are addressed by the clinical team. The prompt list includes five domains: physical and functional well-being, social care and well-being, psychological, emotional and spiritual well-being.

### Ethical considerations

Permission was sought from The Royal Marsden NHS Foundation Trust committee for clinical research with approval granted September 2023 (SE1510). All data was coded and no identifiable reference to the participants held within the data.

### Population

The *retrospective* sample included all patients who were under active consultant-led HNC oncological surveillance who had been treated for primary HNC.

Patients were included in the *prospective* arm if they had (1) a diagnosis of HNC, (2) had received curative HNC treatments and (3) had completed treatment more than 12 months previously. Inclusion criteria required that people were over 18 years old and had adequate linguistic and cognitive function to complete the questionnaire and understand written forms in English. Exclusion included those who were receiving non-standard of care protocols via any clinical trials as well as any patients with suspected or confirmed disease progression or recurrence.

### Data collection

Data collection included patient, tumour and treatment demographics and any recorded information regarding dysphagia. It was noted within the *retrospective* arm, whether medical teams had specifically recorded in the notes when they had asked patients about dysphagia or other swallowing-related toxicity. This was recorded as general enquiry about eating and drinking as patients did not routinely complete any patient reported outcomes. If swallowing difficulties were reported, it was confirmed whether any onward referrals were made (including to SLT).

Within the *prospective* arm, patients were invited to complete the PCI-HaNC-PT prior to a routine medical surveillance appointment. Following completion, they discussed the selected items (representing an unmet need of concern) with a member of the SLT team to guide information provision or signposting to relevant services including other health professionals (HCPs), and/or support with accessing tertiary community services. This review was conducted using the same prompts each time; however, responses were customised according to patient need. People who could not be seen in clinic were followed up via phone call within a week of the appointment.

### Data analysis

Demographic information was recorded to include age, sex, tumour site and staging, as well as previous treatment within both arms. In the case of *retrospective* data collection, the number of patients being reviewed after treatment in a consultant-led clinic for routine cancer surveillance was described as well as the swallowing concerns recorded by patients within medical notes. Within the *prospective* arm, concerns reported using the PCI-HaNC-PT were summarised using descriptive statistics including counts and percentages (categorical data) of symptom items highlighted.

## Results

### Key demographics

#### Retrospective arm

The sample included 52 patients (32 male and 20 female) with a median age of 63 (37–82 years). Tumour sites included oropharynx 60% (*n* = 31), oral cavity 25% (*n* = 13), larynx 13% (*n* = 7) and nasopharynx 2% (*n* = 1). The majority 77% (*n* = 40) were 1–5 years post treatment, 13% (*n* = 7) were 6–10 years post treatment and 10% (*n* = 5) were > 10 years post treatment. Patients were under review more than 5 years post treatment secondary to consultant/patient preference or having been recently re-referred with a change in symptoms.

#### Prospective arm

A total of 103 patients were identified as eligible from weekly monitoring of outpatient clinic lists. In total, 17% (*n* = 18) were excluded for the following reasons: 44% (*n* = 8) were being investigated for osteoradionecrosis (ORN) or active disease; 17% (*n* = 3) had the appointment rescheduled; 28% (*n* = 5) did not attend; and 11% (*n* = 2) were less than 12 months post treatment or did not have treatment for a HNC malignancy. A further 11% (*n* = 11) from the total *prospective* arm were missed in clinic and we were unable to contact them via other means which were attempted (follow-up phone calls and emails on the day or following day of appointment).

A total of 74 patients were included in the analysis. The sample included 45 males and 29 females with a median age of 63 (range 29–86). Tumour sites were as follows: oropharynx 60% (*n* = 44), oral cavity 15% (*n *= 11), parotid 12% (*n* = 9), nasopharynx 8% (*n *= 6) and larynx 5% (*n* = 4). Patients were 1–5 years following treatment in 54% (*n* = 40) of cases, 6–10 years in 30% (*n* = 22) and more than 11 years 16% (*n* = 12). In addition, 27% (*n* = 20) were confirmed to have HPV-related disease. Where data on tumour staging was unavailable, this was due to patients having original treatment outside of our centre.

### Patient concerns

Within the *retrospective* review of patient case notes, documented evidence of questions relating to eating and drinking was noted in 13% (*n* = 7) of cases. Of the seven patients questioned, six reported potential dysphagia with only one of these patients referred for formal assessment to SLT.

**Fig. 1:**
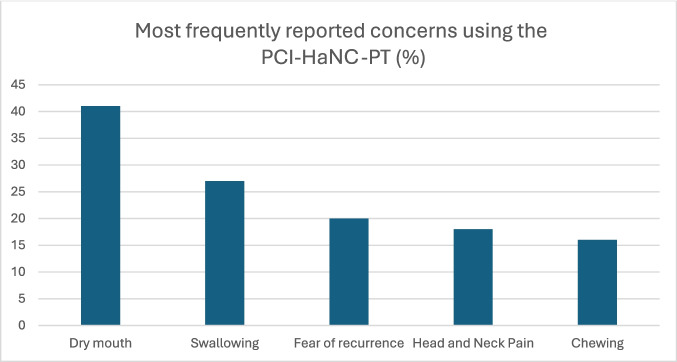
Most frequently reported concerns using the PCI-HaNC-PT

In the *prospective* sample, over 78% (*n* = 58) of patients reported concerns with 1 or more items on the PCI-HaNC-PT and the median number of identified concerns was 5 (range 1–19). The most commonly reported were dry mouth 41% (*n* = 30), dysphagia 27% (*n* = 20), fear of recurrence 20% (*n* = 15), head and neck pain 18% (*n* = 13) and difficulties chewing 16% (*n* = 12) summarised in Fig. 1.

## Discussion

This cross-sectional review sought to evaluate potential unmet needs of patients who were undergoing routine surveillance at a single tertiary referral centre following curative treatment for primary HNC, with a particular focus on swallowing toxicity. While it was the case that a small cohort of patients were reviewed for a longer period of time, secondary to consultant/patient preference, the majority were within 5 years post treatment.

Within the *retrospective* arm, it was found that few patients were asked about their swallowing or swallowing-related symptoms. When dysphagia was identified, this did not signal an automatic referral for SLT assessment. Previously reported evidence suggests that persistent dysphagia among this population is underreported and aspiration pneumonia is recognised as a contributor to non-cancer-related mortality among these cohorts [25, 27].

In keeping with other published studies [10, 28] swallowing-related toxicity was identified within the *prospective* review as a high ongoing priority for patients, with three of the top five concerns related to it.

Our data suggest that while difficulties related specifically to eating and drinking remain a primary concern for patients following treatment, they were not routinely identified at our centre within the current model of follow-up. As the primary function of the surveillance clinic is to identify recurrence, this may not be surprising [29].

However, given our growing understanding of the need to update and personalise approaches to HNC survivorship care, and particularly in the context of the evolving evidence related to late-RAD, there must be an equal focus not only on survival but on survivorship and QoL [13, 29–32]. HPV-related HNC accounts for a growing incidence of cases, particularly in the western world, and there is a known prevalence for dysphagia in these populations [33]. This cohort tends to be younger when diagnosed and as such face specific QoL concerns following treatment end [34].

As the breadth of research in cancer care grows, placing equal importance on survival, functional, and QoL outcomes [35, 36], there must be a mechanism where ongoing concerns can be identified, and patients signposted for support. The expanded and specialist role of members of the multidisciplinary team in HNC has a key role to play with this. While consideration has been given to service re-design and the use of allied health professionals’ expertise to support the diagnostic and prehabilitation parts of the pathway [37], there has been a lack of this same ambition when examining the post treatment system.

Challenges within current surveillance models in the UK have been highlighted previously, and changes to current SOC are likely. These pathway changes have the potential to increase unmet needs. The findings of this paper highlight the need for more personalised and patient-centred methods of survivorship surveillance.

The benefits of patient advocacy and activation have led to positive outcomes with patients being more involved in their recovery and management in other tumour sites [38]. Manne et al.’s work [39] demonstrates how useful the identification of both risk factors and characteristics is when facilitating patient’s HNC survivorship skills and these needs will evolve over time for each individual. By providing a space to discuss concerns related to changes after treatment and specifically, to address functional change over a longer period, patients can be provided with an opportunity to take a facilitated and engaging role in their own management. This approach embodies the principles of realistic medicine, placing emphasis on the role of patient-centred care and shared decision-making to establish the most appropriate interventions for each individual [40].

The use of a prompt list supports patient clinician communication and tailors the clinic appointment to the needs of the patient [41]. By ensuring the consultation and resulting recommendations are focused on patient needs, clinicians can provide a more effective and responsive service. There is ongoing work exploring the feasibility of electronic prompt tools which could potentially allow for the further individualisation and personalisation of services [42]. However, care must be taken to ensure changes do not reduce equity of services and further increase inequalities for those who experience reduced digital literacy [43].

## Limitations

This evaluation took place at a single tertiary referral centre and results are limited by the sample size over a short specified 3-month period. Within the *retrospective* data review, it is possible that in cases where patients were asked about dysphagia and reported no concerns, this was not documented. The case note review was also undertaken by one author, and, as such, the possibility of documentation bias in their interpretation of the notes and data extraction exists. A further limitation of this study is the lack of qualitative data in relation to the patient experience of PROMs to monitor and identify QoL symptoms. The *prospective* arm included only those patients attending their routine appointments and therefore cannot seek to understand the needs and views of those who have chosen not to engage with routine surveillance.

## Conclusion

Within this single-centre evaluation, patients were retrospectively and prospectively reviewed to understand ongoing needs in relation to HNC treatment. Results demonstrated that patients continued to experience swallowing-related toxicity many years after treatment and eating and drinking difficulties remain a high priority for those seeking support. However, patients were not routinely asked about these potential concerns/needs. Within our service, we have commenced work in partnership with people who are experts through experience to build and evaluate services which seek to address ongoing QoL concerns in the context of survivorship. It is anticipated that this will support both patient needs and the need for ongoing robust evidence about late radiation–associated toxicity, including late-RAD, their effects and the development of appropriate services.

## Data Availability

The data that support the findings of this study are available from the corresponding author upon reasonable request.
